# Numerical simulation and design recommendations for prestressed steel stayed columns subjected to static and dynamic effects

**DOI:** 10.1038/s41598-025-14542-7

**Published:** 2025-08-20

**Authors:** Aya AboElnaga, Mohamed Mortagi, Mohamed E. El Madawy, M. Naguib, A. H. A. Abdelrahman

**Affiliations:** Structural Engineering Department, Faculty of Engineering, Mansoura University, Mansoura, 35516 Dakahlia Egypt

**Keywords:** Static and dynamic analysis, Buckling behavior, Prestressed steel stayed columns, Finite element analysis, Design guidelines, Civil engineering, Software

## Abstract

This study investigates the static and dynamic behavior of prestressed steel stayed columns (PSCs), with a particular emphasis on buckling performance under both axial and seismic loading conditions. Unlike prior work that primarily focused on static response or simplified configurations, this research offers a comprehensive investigation encompassing modal analysis and time-history response under seismic excitation, providing valuable insights into the dynamic performance of PSC systems. Advanced finite element (FE) models are developed in ABAQUS using fully automated scripting that defines node coordinates and element connectivity for main columns, cross-arms, and cable stays. These models incorporate geometric and material nonlinearities and are validated against existing experimental and analytical results. A novel design configuration featuring two-level cross-arms is introduced, substantially expanding beyond the conventional single-level systems addressed in earlier studies. Through an extensive parametric study, key parameters such as cross-arm length, cable diameter, and geometric proportions are systematically examined. Based on the numerical findings, new predictive formulas are proposed to estimate the ultimate buckling capacity of two-level PSCs, supporting efficient and resilient preliminary design.

## Introduction

**Fig. 1:**
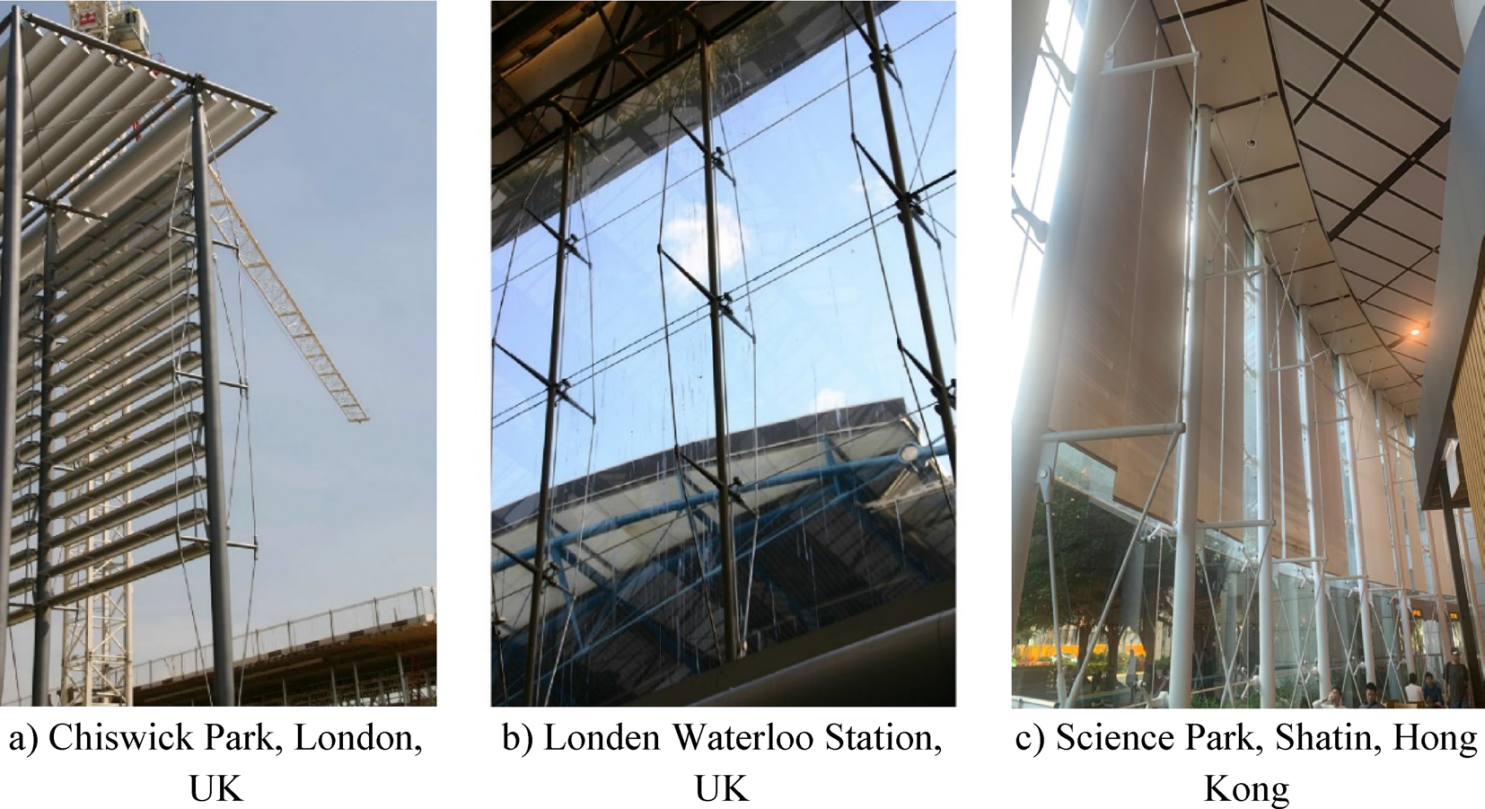
Applications of prestressed steel stayed columns (PSCs).

Prestressed stayed columns (PSCs) represent a considerable advancement in structural engineering, addressing the challenges of low critical buckling loads in slender columns. These columns are a self-equilibrium system with high material efficiency and consist of a slender main column, cross-arm members, and pre-tensioned cable stays, offering aesthetic appeal and structural efficiency. PSCs are particularly useful in tall and wide-span buildings due to their ability to span long distances while maintaining lightweight structures, making them cost-effective for robust load-bearing applications, as illustrated in Fig. 1. Research on PSCs began in the 1960s with key contributions from Chu and Berge^1^ and Temple^2^. Chu^1^ developed a method to calculate the buckling load of struts reinforced with tension ties, setting the stage for optimizing their strength. Temple^2^ introduced cross-arm members and prestressed stays to steel columns, enhancing buckling resistance, with FE analysis revealing the improved performance of this design.

Subsequent research by Hafez et al.^3^, Wong^4^, and Jemah and Williams^5^ advanced the understanding of prestressed stayed columns. Hafez et al.^3^ identified a linear relationship between initial pretension and buckling load ‘prestressing zone of behavior’ but noted significant discrepancies with experimental data, calling for further nonlinear analysis. Wong^4^ examined the effects of initial geometric imperfections on buckling capacity, finding that deviations from straightness reduced both deflection rate and buckling load. Jemah and Williams^5^ proposed a novel column design for space applications, demonstrating reductions in volume and mass compared to conventional designs.

Research on PSCs has advanced significantly, with key contributions addressing stability, buckling behavior, and design optimization. Chan et al.^6^ investigated the second-order stability of prestressed cable-stayed columns with initial imperfections, employing equilibrium polynomial and cable elements. Their parametric study highlighted the effects of imperfections, strut rigidity, column length, and cable size. Saito and Wadee^7–10^ explored the interaction between pretension force and column strength, post-buckling behavior. Moreover, Araujo et al.^11^ used nonlinear FE simulations to examine stayed systems under sudden impact loads. Their analysis revealed how prestress force magnitude and damping influence system performance, evaluating amplification factors against horizontal deflection limits in steel design standards.

Focusing on practical design applications, Osofero^12^ proposed design procedures for PSCs with single cross-arm systems, validated through experimental and FE studies. These procedures considered system geometry, initial prestress, global imperfections, post-buckling effects, and mode interactions, supported by worked examples to illustrate their implementation. Similarly, Serra et al.^13^ conducted an extensive experimental study on 12-m PSCs, performing 44 tests to evaluate compressive strength across varying geometric and material parameters, providing critical data for further numerical and analytical investigations.

To enhance PSC stability, Guo et al.^14^ introduced an innovative buckling-restrained brace (PSC-BRB), incorporating cross-arms and pre-tensioned cables into standard braces to improve structural resilience. Building on this, Li et al.^15–17^ examined stability improvements achieved through pre-tensioned cables and cross-arms, analyzing interactive buckling in multi-branch systems. Their work underscored the significance of initial imperfections in capacity predictions, proposing reduction factors for eccentric loading and evaluating stability in configurations with three cross-arms. Lapira et al.^18^ explored optimal pretension forces in multi-cross-arm systems, demonstrating their effectiveness in resisting buckling while accounting for geometric nonlinearities. Additionally, Yu and Wadee^19^ and Wang et al.^20^ advanced the field by addressing buckling resistance, stability under eccentric loading, cross-arm length optimization, and mode interaction analysis, contributing valuable insights to the design and application of PSCs. Wu et al.^21^ proposed an analytical model for predicting the buckling loads of doubly and mono-symmetric prestressed stayed columns under axial compression, identifying the critical buckling modes without the use of FE analysis.

Recent studies have expanded the understanding of PSCs, with contributions from Hyman and Osofero^22^, Krishnan^23^, and Wu et al.^24^. Hyman and Osofero^22^ investigated the effects of eccentric loading, identifying critical imperfection combinations that significantly reduce load capacity, particularly during interactive mode buckling. Krishnan^23^ emphasized the architectural and structural advantages of cable-stayed columns in buildings, highlighting reduced core sizes and enhanced compression strength through case studies. Zhang and Kim^25^ conducted FE modeling and experimental tests to investigate the behavior of stayed columns. Their analytical study examined the effects of bonded and unbonded cable stays, incorporating second-order effects and consistently accounting for unstrained cable lengths using an energy method formulation. Wu et al.^24^ explored the stability of PSCs under fire conditions, proposing a fire design method based on load reduction. Using steady-state analysis in ABAQUS^26^, they modeled the impact of non-uniform temperature distributions. Liu et al.^27^ introduced and analyzed a novel PSC type—the circular concrete-filled double-skin steel tube (PS-CCFDSST)—through numerical modeling and theoretical derivation, demonstrating its axial compressive behavior.

Further advancing the field, Wu et al.^28^ investigated the buckling behavior of PSCs using FE modeling, incorporating material nonlinearity for the first time in analyzing interactive buckling. Their findings revealed that dual-nonlinearity interactions could diminish the significance of interactive buckling, particularly at high prestressing levels, offering new insights into PSC stability. Wu et al.^29^ numerically examined the buckling and post-buckling behavior of prestressed stayed I-section steel columns, explicitly considering local buckling. The results show that the dominant global buckling mode governs the system’s nonlinear response, typically identified as either Mode 1 (symmetric) or Mode 2 (antisymmetric). When local buckling becomes significant, the influence of stay size, cross-arm length, and prestress level diminishes. The actual optimum prestress closely matches the theoretical prediction in Mode 2-dominated cases but is approximately twice as high in Mode 1-dominated scenarios. Additionally, an artificial neural network (ANN) model was developed to predict the ultimate load capacity.

While the static behavior of PSCs has been extensively studied, their dynamic response, particularly under seismic loading, remains insufficiently understood. Existing design formulas are primarily limited to single-level cross-arm configurations, highlighting the need for further research on multi-level systems. Moreover, most FE analyses to date have not been rigorously validated against experimental results or benchmark studies, especially in dynamic contexts. As a result, there is a lack of robust, validated design equations that accurately capture these effects and offer broad applicability.

There is a critical need for a validated and generalizable model of PSCs capable of simulating structural behavior under both static and dynamic loads across various configurations (e.g., one-, two-, and three-level systems). Additionally, there is a need for comprehensive design equations that incorporate key variables such as stay geometry, cable diameter, and prestressing force.

In contrast to previous studies that focus primarily on single-level PSCs or overlook seismic effects, this study investigates both the static and dynamic behavior of PSCs, with particular emphasis on buckling phenomena and seismic responses. A detailed finite element FE routine developed in ABAQUS is validated against experimental and analytical results from the literature. An extensive parametric study examines the influence of geometric configurations and prestressing forces on PSC performance. Based on the FE results, a predictive design formula for load-carrying capacity is proposed and validated, extending existing research to two-level cross-arm configurations. The study concludes with practical design guidelines to facilitate the use of PSCs in modern structural systems. Furthermore, with the growing emphasis on sustainable construction, the material efficiency and modular reusability of PSC systems present promising opportunities for environmentally conscious structural solutions^30,31^.

## Finite element modeling

The FE capabilities of ABAQUS are utilized to comprehensively analyze the static and dynamic behavior of PSCs. An automated modeling framework is developed using Python scripting to efficiently generate and manage the entire model setup. This framework defines node coordinates, element connectivity for the main column, cross-arms, and cable stays, and accommodates complex configurations such as multi-level PSCs and stayed frames. Critical modeling parameters—including geometry, mesh refinement, material properties, loading schemes, boundary conditions, and analysis types—are systematically integrated into the automated process to ensure consistency and accuracy across simulations.

### Modeling parameters

To investigate the behavior of PSCs under various buckling and failure modes, a line-based FE model (LFEM) is employed. The LFEM utilizes beam elements (B23 for 2D analysis and B32 for 3D analysis) to model the column and cross-arm, while B32 beam elements were adopted for the 3D models. These were selected due to their efficiency and suitability in modeling slender beam–column elements under axial and flexural loading. For the stays, truss elements (T2D2 for 2D analysis and T3D2 for 3D analysis) with “No compression” behavior are used, enabling tension-only response. This approach allows each cable to be modeled individually, ensuring accurate representation of its response under different loading conditions.

**Fig. 2:**
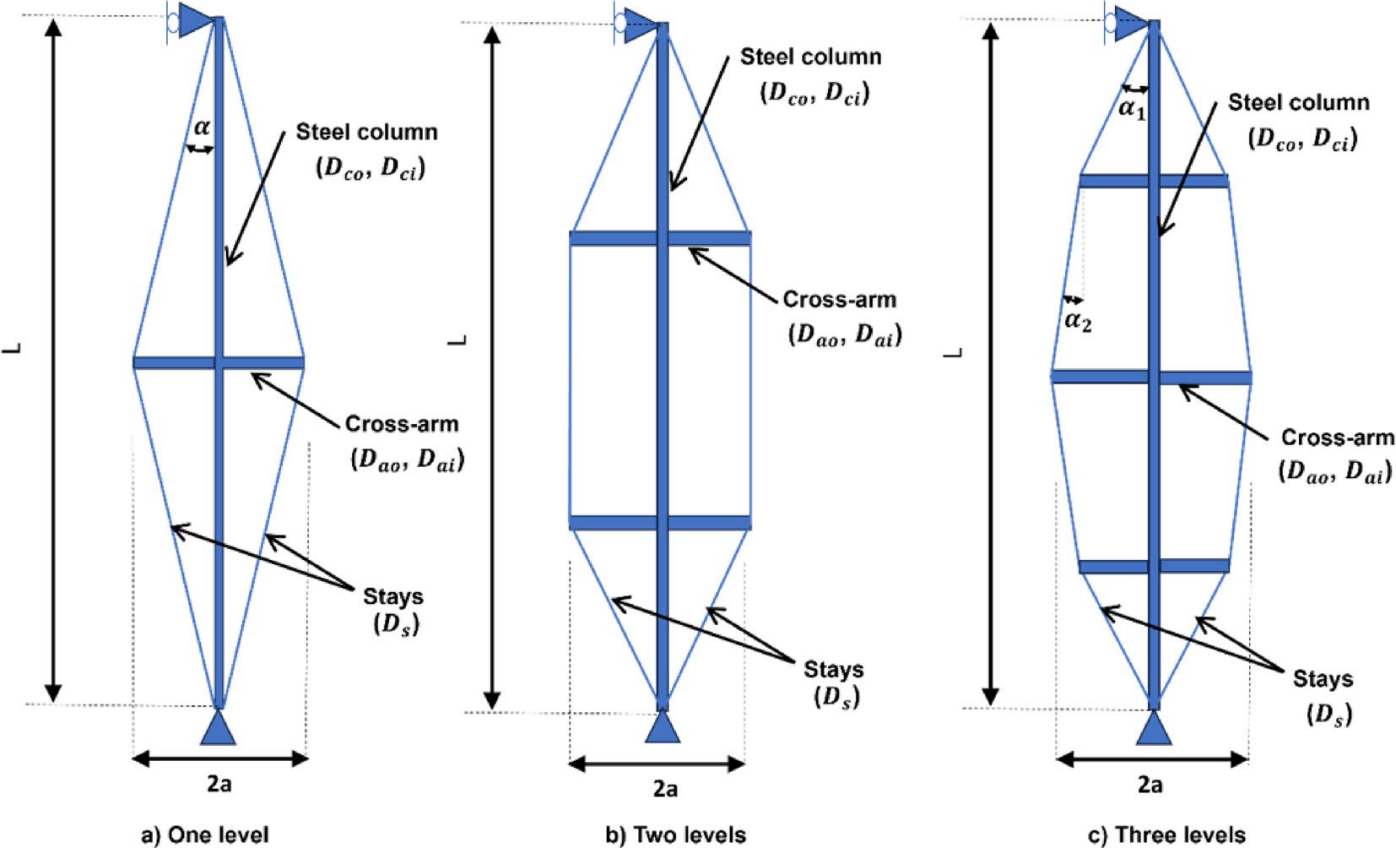
Geometric configurations of PSCs.

The geometric and material properties of PSCs play a critical role in defining their structural behavior. Key geometric parameters include the column length (${L}_{c}$) and cross-arm length ($a$). The cross-sectional dimensions are defined by the outer and inner diameters of the column (${D}_{co}$, ${D}_{ci}$), the cross-arm (${D}_{ao}$, ${D}_{ai}$), and the stay diameter (${D}_{s}$). The material properties are defined by the Young’s modulus of the column (${E}_{c}$), cross-arm (${E}_{a}$), and stays (${E}_{s}$). In cases involving multi-level cross-arm configurations or varying arm geometries, a comprehensive set of dimensional parameters is employed to accurately capture the structural geometry and behavior. An illustrative example of geometric configuration is presented in Fig. 2.

The material properties of PSCs, particularly those of the steel components, play a critical role in structural analysis. The elastic properties, characterized by the modulus of elasticity ($E$) and Poisson’s ratio ($\nu$), determine the material’s stiffness and response to deformation. The yield stress represents the inelastic behavior (${F}_{y}$), where plastic deformation begins. In this study, an elastic-perfectly-plastic constitutive model is employed for the steel column. This approach effectively captures both the elastic and plastic phases of deformation, enabling a detailed and accurate analysis of the PSCs’ response under various loading conditions. The meshed size of the main member and crossarms is 50 mm, and each stay is meshed as an individual element.

### Boundary conditions and loading scenarios

The connections between the stays and the main column, as well as between the stays and cross-arms, are modeled as ideal pins using coupling constraints to ensure consistent displacement behavior between the stays and the cross-arms. This approach enforces equilibrium at the connection points and accurately simulates the interaction between these components.

The connections between the cross-arms and the main column are modeled as rigid for both static and dynamic analyses. The boundary conditions at the reference nodes on both ends are defined by the degrees of freedom (Ux, Uy, Uz, Rx, Ry, Rz), enabling the specification of arbitrary constraints within the proposed automated FE routine. Where Ux, Uy, and Uz denote translational displacements along the *x*-, *y*-, and *z*-axes, respectively, and Rx, Ry, and Rz represent rotational displacements about the corresponding axes.

For the static analysis, a concentrated load is applied at the top of the column. In the dynamic analysis, the system is subjected to seismic excitation based on an arbitrary time history scenario, with the El Centro earthquake applied in the X direction at the column base as a reference excitation. A lumped mass is assigned at the column’s center, and the time history amplitude is provided in tabular form. Direct modal damping is used to represent member viscosity, incorporating a specified critical damping ratio and time step (Δt). The member’s self-weight is excluded from dynamic analysis to isolate the effects of external loading conditions.

### Prestress

**Fig. 3:**
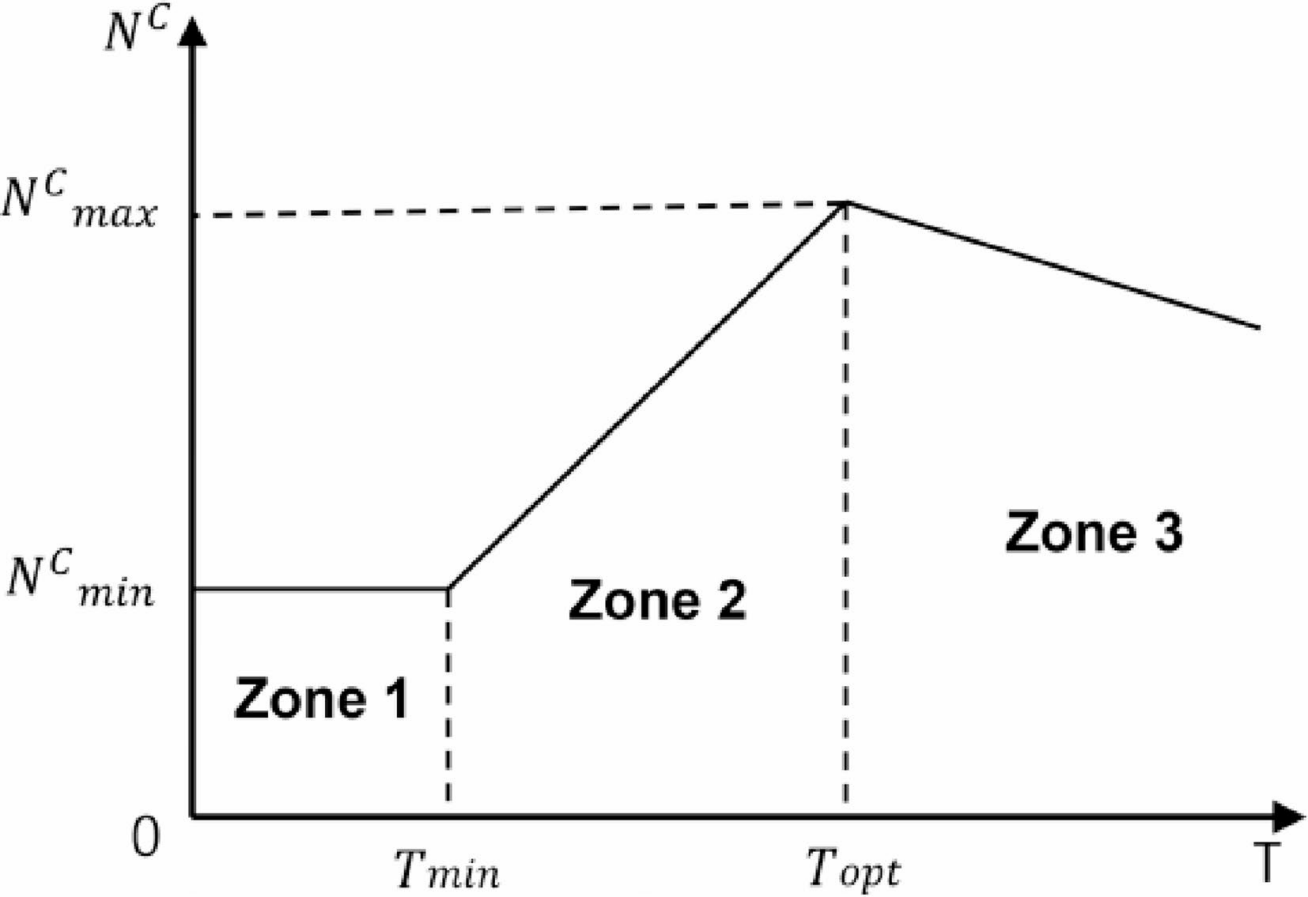
Critical load *N*^*C*^ versus the initial prestress *T* presented with zone distinction.

The effect of prestress in cables is essential to the behavior and stability of PSCs. The relationship between prestressing force and critical buckling load offers a useful estimate for determining the required prestress. As shown in Fig. 3, understanding these variations in critical loads is crucial for optimizing the design and performance of PSCs. Initially, a prestressing force is applied to the cable stays to improve the PSCs’ load-bearing capacity. Figure 3 illustrates the correlation between critical buckling load and varying prestress levels, as observed in previous studies^3^.

Zone 1 represents minimal prestressing force, where T < ${T}_{min}$. In this zone, the critical buckling load of the unstayed main column, also known as the Euler critical buckling load (${P}_{E}$), is reached under axial loading. In Zone 2, the prestressing force increases to $T$ = [${T}_{min}$, ${T}_{opt}$], enhancing the column’s load capacity beyond the Euler load. However, at buckling initiation, the stays lose their tensile force. Zone 3 involves a higher prestressing force, ensuring residual tension in the stays when buckling begins. The optimal prestressing force ${T}_{opt}$, positioned at the boundary between Zones 2 and 3, maximizes the critical buckling load (${N}_{max}^{c}$). Hafez et al.^3^ provided the expression for this optimal force in single bay stayed columns as follows:

1 $$T_{opt} = N_{T = 0} \frac{{C_{1} }}{{C_{2} }}$$

2 $$T_{\min } = C_{1} N_{\min }^{c}$$

3 $$N_{min}^{c} = \left\{ \begin{gathered} N_{E } \quad \;\left( {mode 1} \right) \hfill \\ 4N_{E} \quad \,\left( {mode 2} \right) \hfill \\ \end{gathered} \right.$$

4 $$C_{1} = \frac{\cos \alpha }{{2K_{c} \left( {\frac{1}{{K_{S} }} + \frac{{2\sin^{2} \alpha }}{{K_{a} }} + \frac{{n\cos^{2} \alpha }}{{K_{c} }}} \right)}}$$

5 $$C_{2} = 1 + \frac{{n cos^{2} \alpha }}{{K_{c} \left( {\frac{1}{{K_{S} }} + \frac{{2sin^{2} \alpha }}{{K_{a} }}} \right)}}$$

where ${N}_{T=0}$ represents the buckling load when the initial pretension $T$ equals zero. The parameters ${C}_{1}$ and ${C}_{2}$ are determined by the geometric configurations and material properties of the PSC. ${K}_{S}$, ${K}_{a}$, and ${K}_{c}$ represent the axial stiffness of the stays, cross-arms, and main column, respectively. $\alpha$ is the angle between the main column and the stays. $n$ is a parameter related to the typology of the stayed column ($n=1$ for plane stayed column and $n=2$ for spatial stayed column).

In the proposed FE framework, the prestressing effect for inclined cables is modeled using an initial temperature load. The prestressing step is performed before the loading step, where the temperature variation induces thermal strain ($\varepsilon =\phi \Delta \tau$) and generates the corresponding cable force. The cables were prestressed by using the thermal contraction of steel as it cools, to produce an internal tensile force. Here, $\phi$ represents the thermal expansion coefficient, and $\Delta \tau$ denotes the temperature change. As the cable temperature decreases, the cable force increases; hence, a negative temperature difference is applied to the prestressed cables, as follows^32^:

6 $$\Delta \tau = - \frac{\varepsilon }{\phi } = - \frac{\sigma }{{\phi * E_{s} }} = - \frac{P}{{\phi * E_{s} * A}}$$

where ${E}_{s}$ is the modulus of elasticity of the cable, A is the cross-sectional area of the cables, and *P* is the prestressing force. In the FE models, the optimum prestressing force (${T}_{opt}$), and the corresponding temperature change ($\Delta\uptau$) are applied to simulate the prestressing effect accurately.

### Type of analysis

**Fig. 4:**
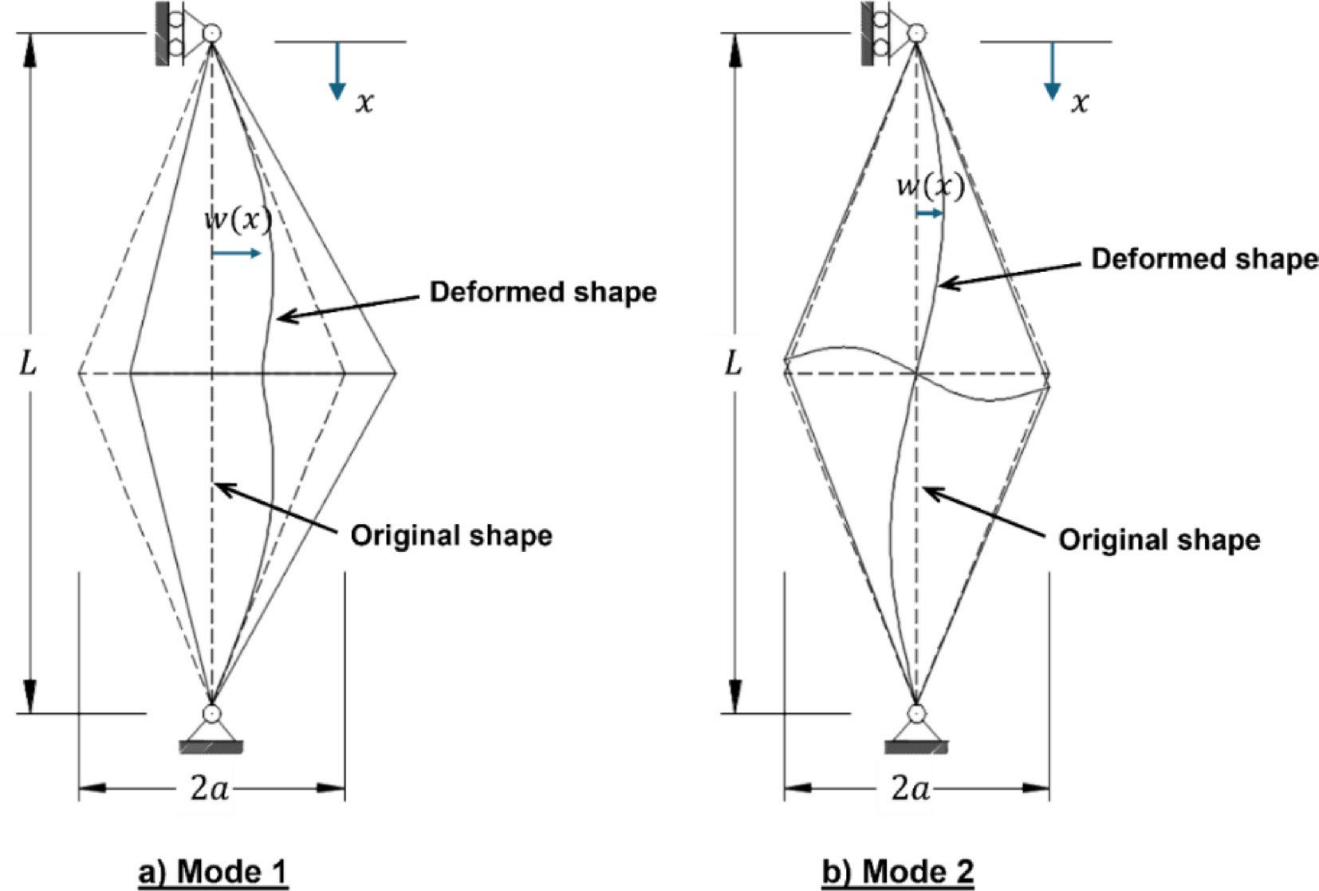
Buckling mode shapes of PSCs; (**a**) Mode 1 (symmetric), and (**b**) Mode 2 (antisymmetric).

Eigenvalue buckling analysis was performed on an idealized (“perfect”) geometry to identify the potential buckling modes of prestressed steel stayed columns, as illustrated in Fig. 4. This idealization refers to a structure with no initial imperfections, residual stresses, or nonlinear material properties. All materials are assumed to behave in a linear-elastic manner, and loading is applied quasi-statically. Although this analysis does not replicate real-life conditions, it serves as a first-order approximation for predicting elastic buckling behavior.

Eigenvalue buckling analysis—also known as linear buckling analysis—is a numerical method used to estimate the critical buckling load of a structure under ideal, unfactored conditions^33^. It determines the theoretical load at which a structure loses stability by solving an eigenvalue problem derived from the linearized equilibrium equations. This technique helps identify buckling mode shapes and their corresponding load multipliers, thereby revealing the most critical instability mechanisms in slender structural systems.

In this study, eigenvalue buckling analysis serves as a preliminary step in assessing the global stability of PSCs. It provides insights into dominant buckling modes, which inform and guide the subsequent nonlinear and dynamic analyses presented in the later sections of the paper. Mode 1 (symmetric) represents uniform bending along the column axis, while Mode 2 (antisymmetric) depicts opposing deformation patterns across the column’s axis.

This preliminary analysis is essential for predicting the column’s failure mode shapes, providing a reference for applying initial imperfection shapes in subsequent nonlinear static buckling analysis. The nonlinear analysis incorporated both geometric and material nonlinearities, offering a comprehensive insight into the column’s nonlinear buckling behavior and enabling a more accurate assessment of load-bearing capacity under realistic conditions.

In addition to examining static and buckling behavior, vibration and modal frequency analyses are critical to accurately determining damping coefficients, contributing to a more comprehensive design for PSCs. Time history analysis further enhances this assessment by enabling the evaluation of structural responses to dynamic loads, such as seismic activity, wind forces, and impact loads. The proposed FE framework is well-equipped to conduct these advanced analyses using implicit dynamic methods, ensuring robust and precise modeling under diverse loading scenarios.

**Fig. 5:**
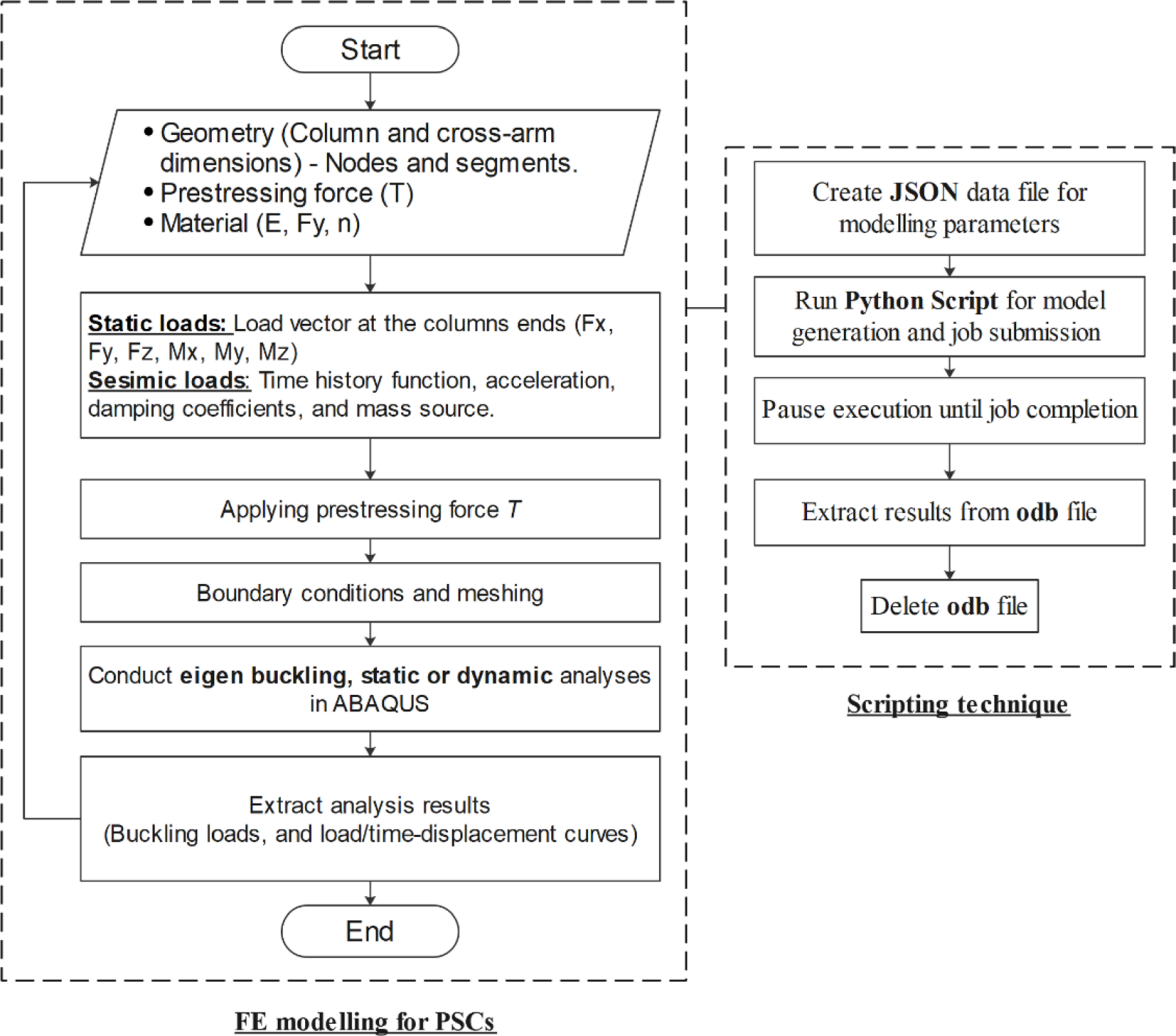
Flowchart for the developed FE framework for PSCs.

Dynamic implicit analysis is especially effective for evaluating structural response to seismic events, as it employs an implicit integration scheme that prioritizes stability and accuracy over extended simulation periods. This approach solves the equations of motion with a focus on the system’s inertia, enabling the FE model to capture essential dynamic properties, including natural frequencies, mode shapes, and transient responses during realistic dynamic events^34^. A comprehensive flowchart of the FE routine is depicted in Fig. 5, illustrating the scripting process that includes model generation, job submission, and extraction of analysis results across diverse loading conditions and analysis types. Although the geometry of the single- and multiple-cross-arm PSCs is relatively simple, the automated script can generate any complex geometry, including models with multiple branches and cross-arms. Using joint coordinates and connectivity definitions, the column system, along with stays and arms, can be defined while accounting for various connection types and material properties. The applied loads may be static, dynamic, or seismic. Moreover, the script can handle column definitions using either line or shell elements, which is particularly important when local and global buckling behavior is to be studied. Composite columns, such as CFSTs, can also be generated using the same framework. This level of automation allows extensive parametric studies and facilitates design investigations efficiently. The JSON file serves as an input file containing key modeling parameters for the proposed framework (e.g., joint coordinates, connectivity, material properties, loads, boundary conditions, type of analysis, etc.)

## Verification and validation of FE models

The purpose of this section is to validate the proposed framework for automating the generation of the developed FE model for various types of analysis and to verify the FE results through three key aspects: 1) buckling loads, 2) mode shapes, and 3) transient responses. Two specific examples are presented to demonstrate the effectiveness of the framework: 1) static nonlinear buckling analysis and 2) transient response analysis.

### Static nonlinear buckling analysis

This example investigates the results of static nonlinear buckling analysis on a prestressed steel stayed column generated using the proposed framework. It is important to note that the maximum buckling load (${N}_{max}$) depends on factors such as stay diameter, initial tension force, and imperfections. For the current model, the parameters specified in^7^ are utilized, including a column length of ${L}_{c}=3050$ mm and a cross-arm length of $a=305$ mm. The outer and inner diameters of the steel column, along with the corresponding diameters for the cross-arms, are 38.1 mm and 25.7 mm, respectively. Additionally, the diameter of the stays is 4.8 mm. Young’s modulus for both the steel column and cross-arm is set at 201 GPa, while that for the stays is 202 GPa.

**Fig. 6:**
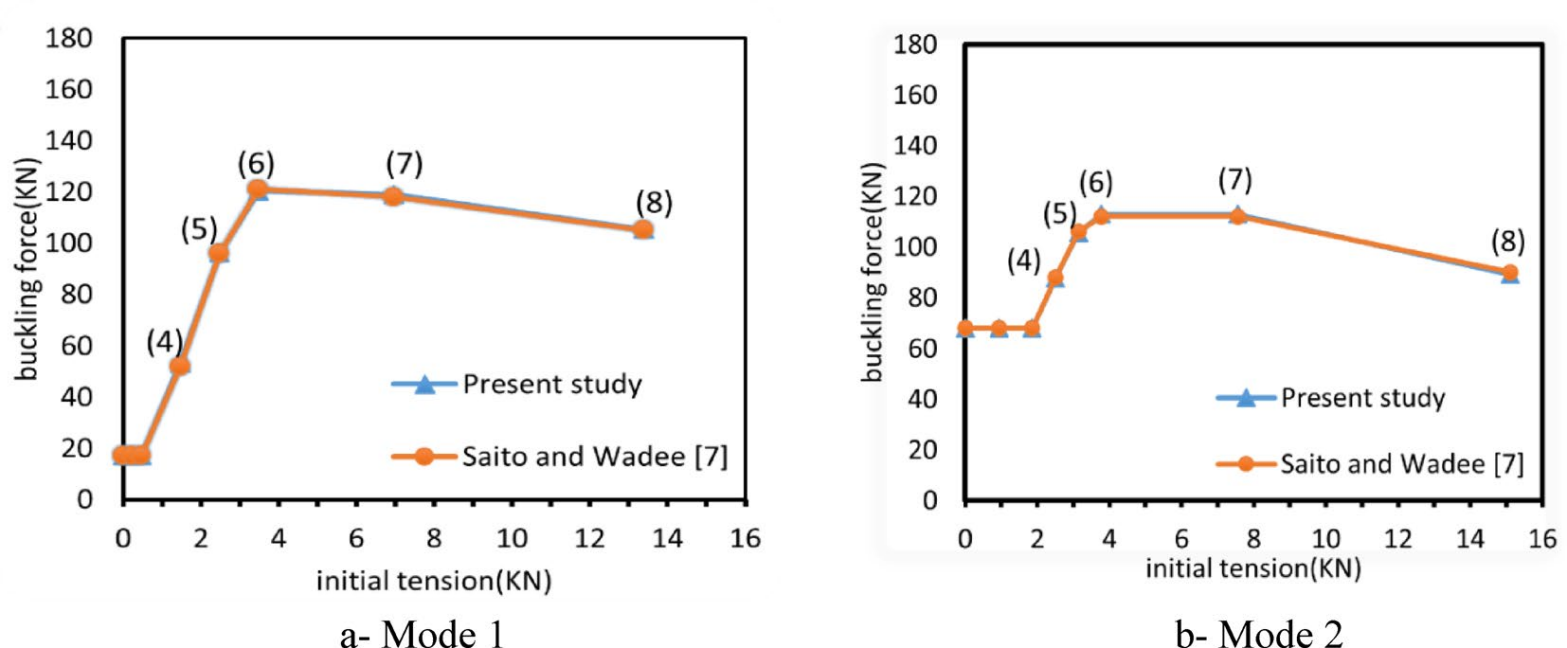
Critical buckling loads against the initial prestress force.

The critical buckling loads to the initial prestress force are illustrated in Fig. 6. Eight points are selected from each diagram to analyze the variations in buckling response as the initial prestress force (T) changes, as detailed in Table 1. According to^7^, the imperfection amplitudes for the anti-symmetric (Mode 2) and symmetric (Mode 1) shapes are set at 0.02 ($${L}_{c}/14142)$$ and 0.03 ($${L}_{c}/10000)$$, respectively. These values are sufficiently small to closely approximate an ideal system. The results show strong agreement, with the deviation not exceeding 0.80% (calculated as: Diff % = (Present study − Past study)/Past study), and nearly identical values were observed.

**Table 1:** Selected points for the buckling investigation_._

Point	Initial prestress T Criterion expression	Initial prestress (kN)	Buckling force (kN)			Initial prestress (kN)	Buckling force (kN)		
		Mode 1	Saito and Wadee^7^	Present study	Diff. %	Mode 2	Saito and Wadee^7^	Present study	Diff. %
1	0	0.00	17.2	17.2	0.00	0.00	68	68	0.00
2	${T}_{min}/2$	0.23	17.2	17.2	0.00	0.93	68	68	0.00
3	${T}_{min}$	0.46	17.2	17.2	0.00	1.86	68	68	0.00
4	(${T}_{opt}$ − *T*_*min*_)/3 + ${T}_{min}$	1.47	52	52.25	0.48	2.50	88	87.8	-0.20
5	2(${T}_{opt}$ − ${T}_{min}$)/3 + ${T}_{min}$	2.48	96	96.08	0.00	3.14	106	105.6	-0.30
6	${T}_{opt}$	3.48	121	120.3	-0.50	3.78	112	112.9	0.80
7	$2{T}_{opt}$	6.97	118	118.9	0.70	7.55	112	112.9	0.80
8	4 ${T}_{opt}$	13.93	105	105.3	0.20	15.10	90	89.2	0.80

**Fig. 7:**
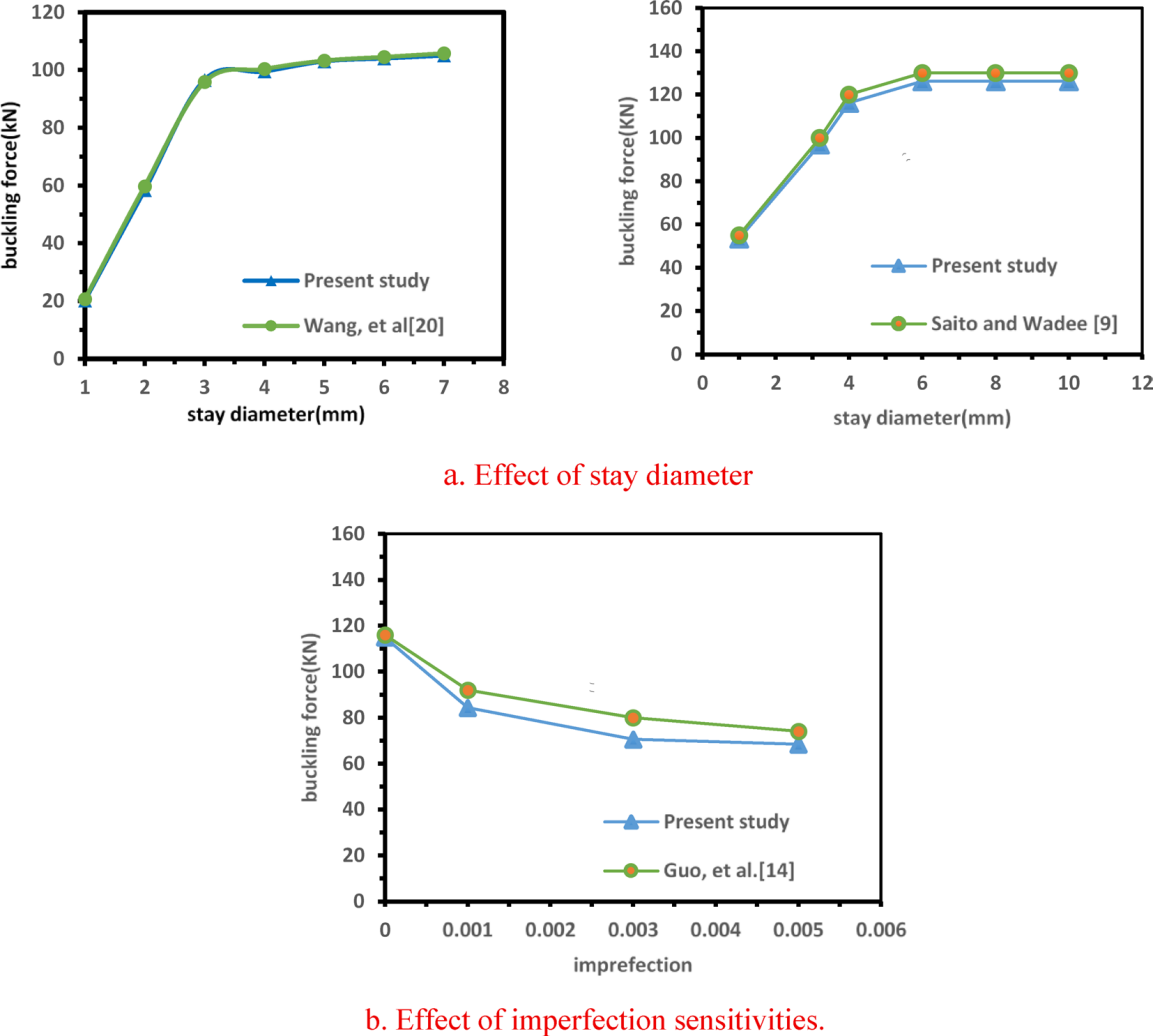
Effect of stay diameter and imperfection sensitivities.

To ensure the reliability and credibility of the proposed models, the imperfection values versus buckling loads curve from^14^ Fig. 7b, along with the corresponding stay diameters and buckling loads in^9,20^ for three levels of cross-arms, are compared against the FE results, as illustrated in Fig. 7a for mode 1.The results show that remarkable alignment does not exceed the percentage of 8% This close agreement indicates that the proposed model can effectively and accurately simulate the behavior of PSCs for nonlinear buckling analysis.

### Three-dimensional dynamic modeling

**Fig. 8:**
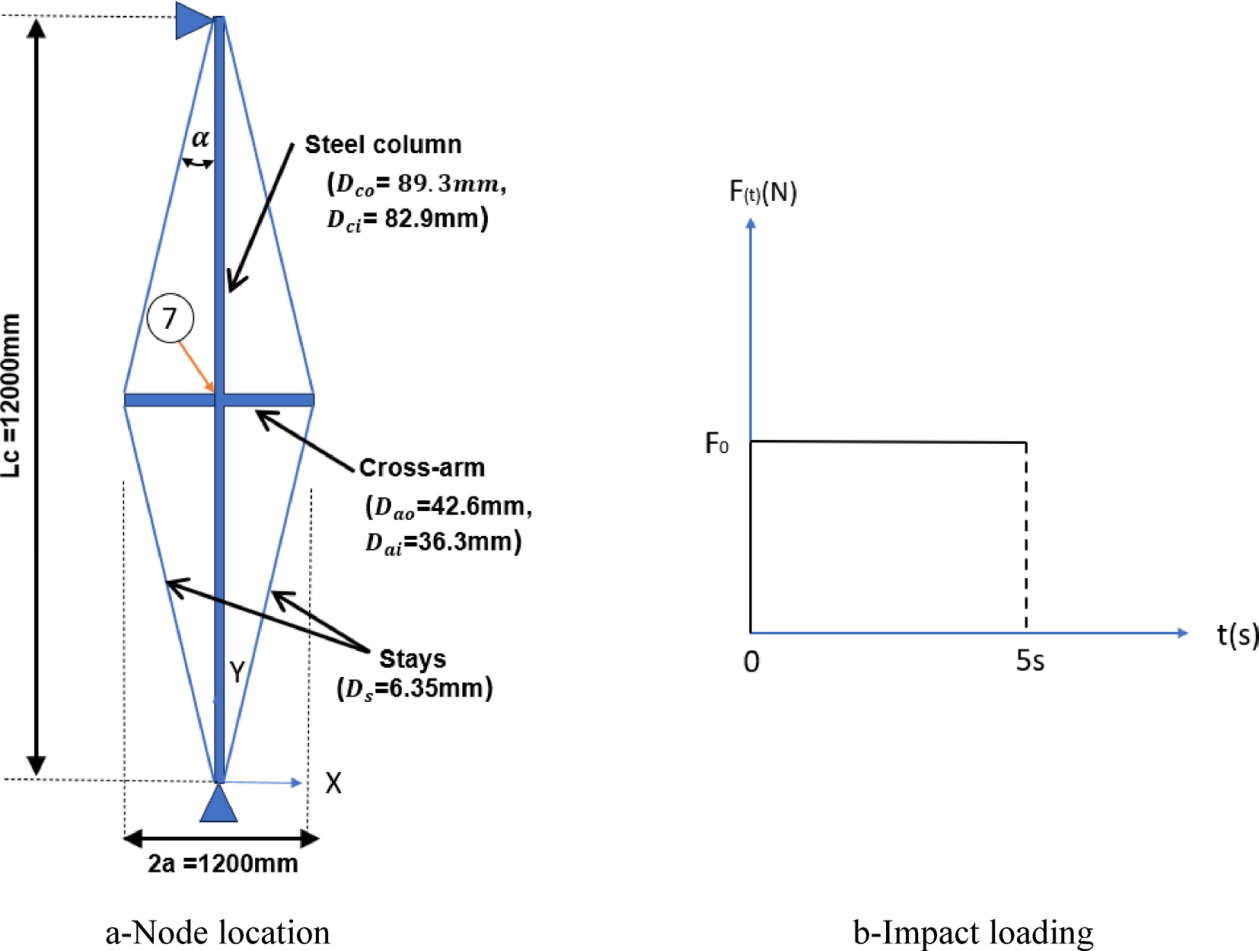
Prestressed stayed steel column: node location and impact loading.

The prestressed steel stayed column was modeled using cables and bars as stays, with a non-linear dynamic analysis employed to simulate an impact load scenario. A rectangular pulse was applied at the top of the structural system, representing a sudden load over a duration of five seconds. The analysis investigated the effects of dynamic loads, ranging from 10 to 80% of the stayed column’s capacity, both with and without prestress forces, while incorporating structural damping. A time step of 0.001 s (Δt = 10^−3^ s) was adopted for precise analysis shown in Fig. 8.

The column had a length of 12,000 mm, and the cross-arms extended 600 mm from the main column. The outer and inner diameters of the column were 89.3 mm and 82.9 mm, respectively. For the cross-arms, the outer diameter was 42.6 mm, and the inner diameter was 36.3 mm. The stays had a diameter of 6.35 mm. The Young’s modulus was 201 kN/mm^2^ for the column and cross-arms, while the stays had a slightly higher modulus of 202 kN/mm^2^. These detailed dimensions and material properties were chosen to ensure the model accurately replicated the dynamic behavior of the prestressed stayed column under impact loading conditions^11^. An 8 mm amplitude sinusoidal initial imperfection was introduced in the numerical model to be compatible with the tests measured imperfections.

Table 2 illustrates the dynamic vertical displacements (along the Y-axis) at node 7 for varying magnitudes of applied sudden loads, ranging from 10 to 80% of the system’s critical load. These loads were applied to the top of the column, with a comparison between the results of Araujo and Santos da Silva^11^ and the findings of the current investigation. The results show that remarkable alignment does not exceed the percentage of 4.50%. The small deviations observed are within an acceptable margin of numerical tolerance and can be attributed to slight differences in idealization or meshing strategies. Overall, the comparison validates the finite element model and confirms that it can accurately capture the static and dynamic behavior of prestressed stayed columns.

**Table 2:** Vertical displacements along the y-axis for structural systems without and with prestress forces.

P_cr_ = 21086.8 N	Without Prestress forces			With Prestress forces		
	Displacement along the vertical y-axis (mm)			Displacement along the vertical y-axis (mm)		
	Araujo, Santos da Silva^11^	Present study	Dif. %	Araujo, Santos da Silva^11^	Present study	Dif. %
P_cr_ 10%	0.13	0.13	0.00	0.78	0.78	0.00
P_cr_ 20%	0.27	0.26	-3.70	0.96	0.96	0.00
P_cr_ 30%	0.40	0.39	-2.50	1.15	1.16	0.08
P_cr_ 40%	0.54	0.52	-3.70	1.34	1.34	0.00
P_cr_ 50%	0.67	0.64	-4.50	1.53	1.54	0.65
P_cr_ 60%	0.80	0.79	-1.25	1.72	1.72	0.00
P_cr_ 70%	0.94	0.92	-2.12	1.91	1.92	0.50
P_cr_ 80%	1.07	1.04	-2.80	2.09	2.09	0.00

## Parametric study

**Fig. 9:**
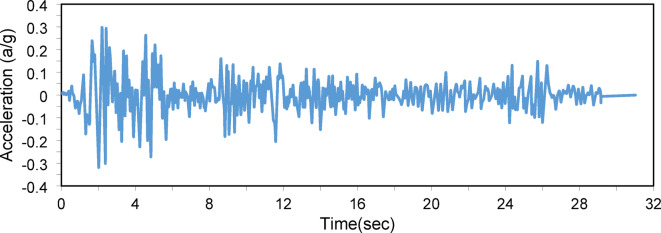
EI-Centro Waves functions applied for the column.

For the dynamic analysis, the columns were subjected to seismic excitations using the El Centro earthquake wave functions, as illustrated in Fig. 9. The El-Centro earthquake is a well-documented seismic event and is widely used in structural engineering studies due to its comprehensive ground motion data. This seismic record is characterized by significant peak ground accelerations and frequency content that challenge the stability of structures, making it a reasonable benchmark for assessing dynamic performance. The seismic load, applied at the base of the column in the X direction, was modeled with a lumped mass at the center of the column. Structural damping was represented by direct modal damping with a critical damping ratio of 0.005 and a time step (Δt = 0.02) for the first three modes. The damping matrix was constructed using Rayleigh damping, targeting proportional damping for the fundamental frequencies. While this approach ensures computational efficiency, it may not fully capture the coupled vibration modes typical of hybrid structures. Hybrid structures, such as PSCs with cross-arms and cables, often exhibit non-classical damping behavior due to varying material and component properties. This leads to mode coupling, where classical damping assumptions may fail to represent the dynamic response accurately. Non-classical damping approaches, which incorporate coupling effects, provide a more realistic representation of the damping forces acting on the system. The self-weight of the structural members was also incorporated into the dynamic analysis.

To account for viscous damping within the system and simplify the evaluation of distributed damping coefficients along member lengths, the Rayleigh damping model^35^ is employed in the current study. The damping matrix $[C]$ is expressed as:

7 $$\left[ C \right] = \beta_{1} \left[ M \right] + \beta_{2} \left[ K \right]$$

where ${\beta }_{1}$ and ${\beta }_{2}$ are proportional coefficients associated with the system’s mass $[M]$ and stiffness $[K]$, respectively. These coefficients can be calculated based on a first-order elastic modal analysis using the following formulas:

8 $$\beta_{1} = \frac{{4\pi \left( {\varepsilon_{1} \omega_{1} - \varepsilon_{2} \omega_{2 } } \right)}}{{\left( {\omega_{1}^{2} - \omega_{2}^{2} } \right)}}$$

9 $$\beta_{2} = \frac{{\omega_{1} \omega_{2 } \left( {\varepsilon_{1} \omega_{1} - \varepsilon_{2} \omega_{2 } } \right) }}{{\pi \left( {\omega_{1}^{2} - \omega_{2}^{2} } \right)}}$$

A preliminary modal analysis was first performed to extract the natural frequencies of the structure. Where, ${\omega }_{1}$ and ${\omega }_{2}$ are the natural frequencies of the first and second modes, respectively, and ${\varepsilon }_{1}$ and ${\varepsilon }_{2}$ are the damping ratios equal to 0.005 (0.5%)^11^ for the first and second modes. In this study, ${\omega }_{1}=2.8$ rad/s and ${\omega }_{2 }=39.23$ rad/s then, the proportional coefficients are taken as ${\beta }_{1}=0.05$, ${\beta }_{2}=0.002$.

The results of the dynamic analysis are presented in terms of lateral displacements at the midpoint of the column versus time, providing insights into the system’s dynamic response under varying conditions.

### Cross arm length

The cross-arm length ($a$), which influences the stay angle ($\alpha$) with the column axis, affects both buckling load and dynamic response. In this study, the cross-arm length ratio ($2a/L$) ranges from 0.07 to 0.28, corresponding to inclination angles between 4° and 15°. The column length is ${L}_{c}= 3050$ mm, with a cable diameter of 4.8 mm, and single-level cross-arms featuring four branches. The modeling parameters are as follows: ${D}_{co}$ = 38.1 mm, ${D}_{ci}$ = 25.4 mm, ${D}_{ao}$ = 38.1 mm, ${D}_{ai}$ = 25.4 mm, ${E}_{c}$ = 201 GPa, ${E}_{a}$ = 201 GPa, and ${E}_{s}$= 202 GPa.

**Fig. 10:**
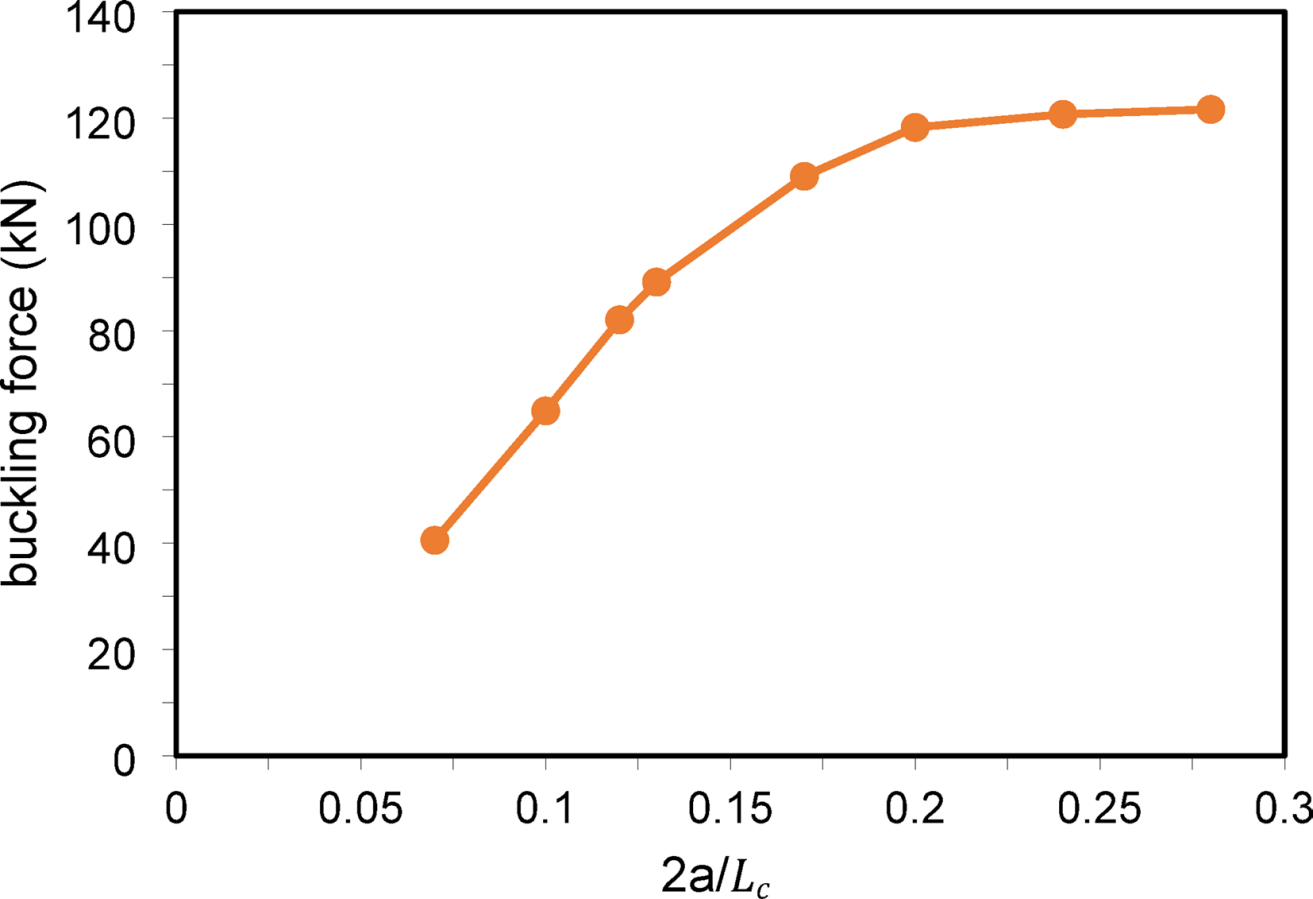
Static buckling force with cross-arm length.

**Fig. 11:**
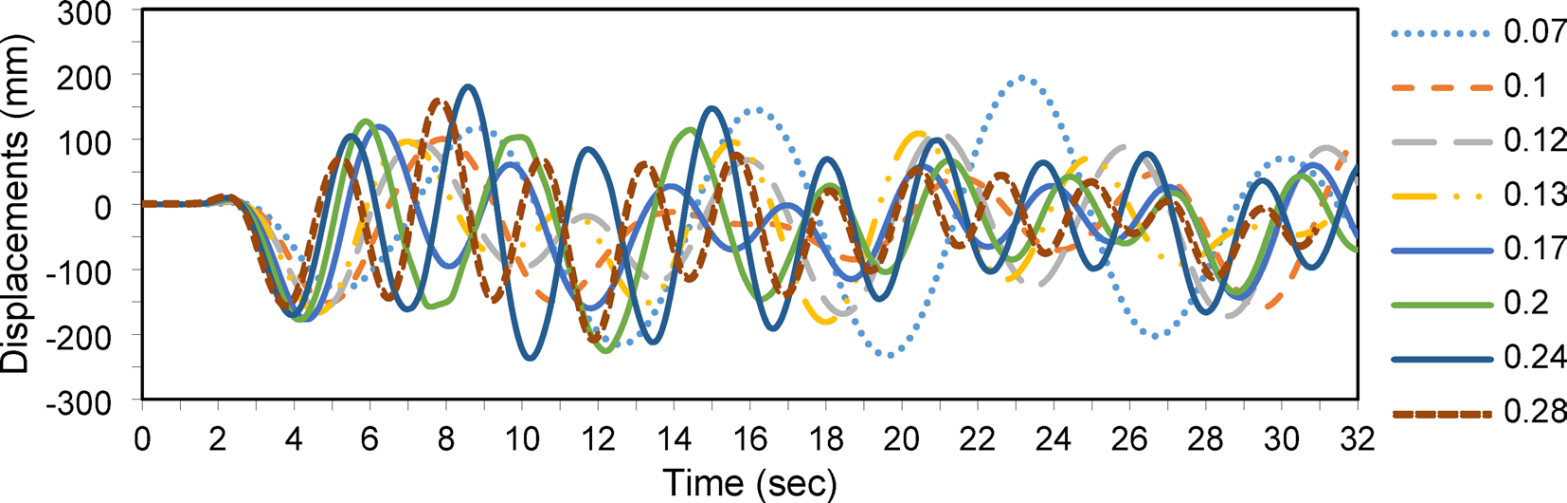
Dynamic responses of PSCs under EI-Centro Waves corresponding to 2a/L.

Figure 10 shows the buckling load versus the cross-arm length ratio, with a prestressing force of ${T}_{opt}$. The buckling load increases with the cross-arm length up to 2a/L = 0.2 (α = 11.3°), after which further length increases yield minimal improvements. For seismic analysis Fig. 11, peak displacements decrease as cross-arm length increases, while response fluctuations intensify. Displacements reduce gradually as 2a/L grows, suggesting enhanced damping and energy dissipation. In conclusion, increasing the cross-arm length significantly improves both static and dynamic performance up to a point beyond which the benefits plateau.

### Cable diameter and prestressing force

In this example, the effect of the stay diameter (${D}_{s}$), which impacts the applied prestressing force, is examined. The diameter varies from 2 to 10 mm, while other geometric parameters are held constant: column length = 3050 mm, cross-arm length = 305 mm, with a single cross-arm level and four branches. The material properties are ${D}_{co}$ = 38.1 mm, ${D}_{ci}$ = 25.4 mm, ${D}_{ao}$ = 38.1 mm, ${D}_{ai}$ = 25.4 mm, ${E}_{c}$ = 201 kN/mm^2^, ${E}_{a}$ = 201 kN/mm^2^, and ${E}_{s}$ = 202 kN/mm^2^. Prestressing force (${T}_{opt}$) is proportional to the stay diameter, as defined in Eqs. (1–5). Increasing the stay diameter increases the sectional area and, consequently, the prestressing force.

**Fig. 12:**
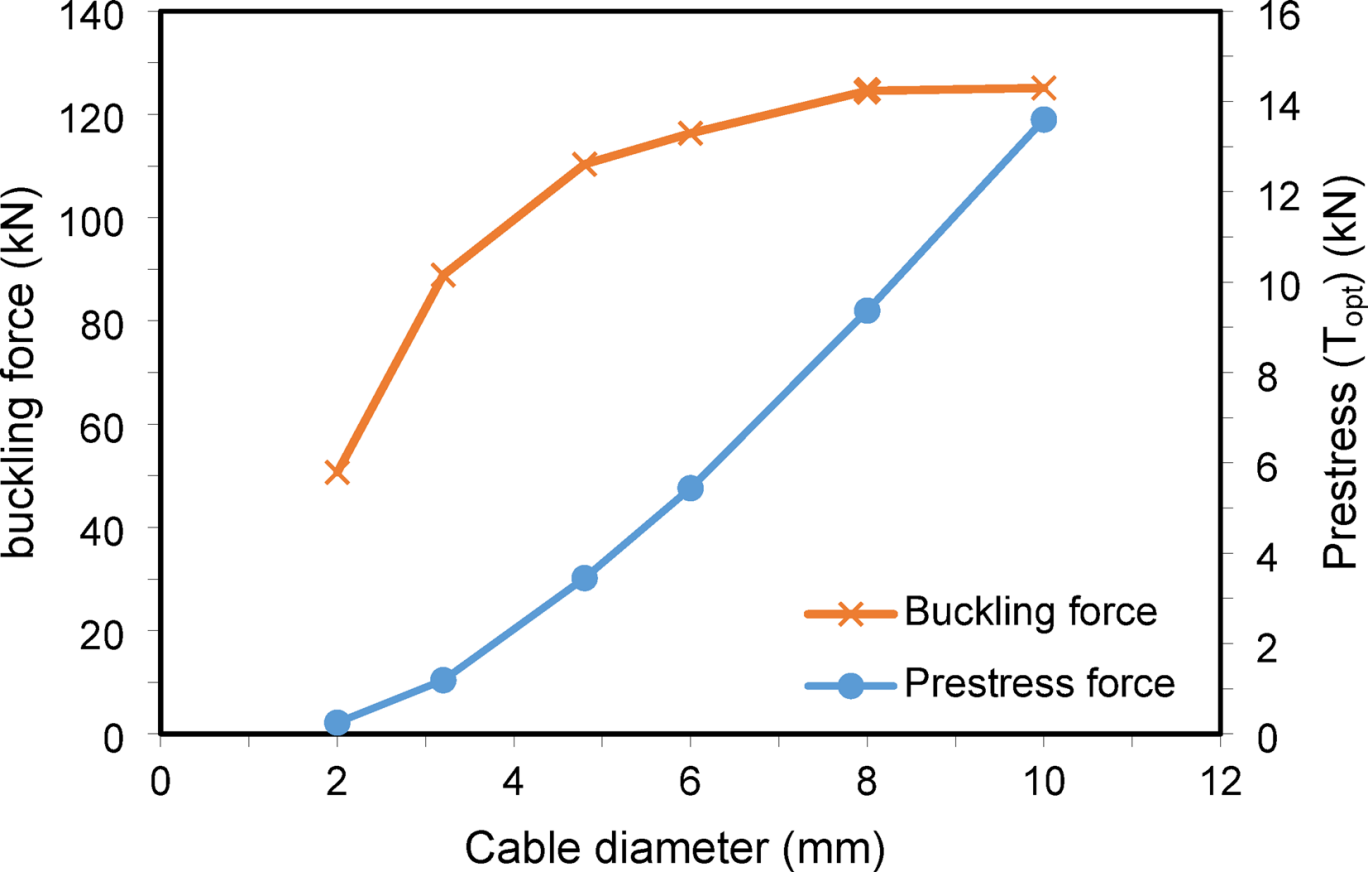
Static buckling force with cable diameter.

Figure 12 illustrates that the buckling load increases with the stay diameter up to ${D}_{s}$ = 8 mm. Beyond this point, further increases in diameter and prestressing force do not affect the buckling load, aligning with the Zone 2 boundary shown in Fig. 3.

**Fig. 13:**
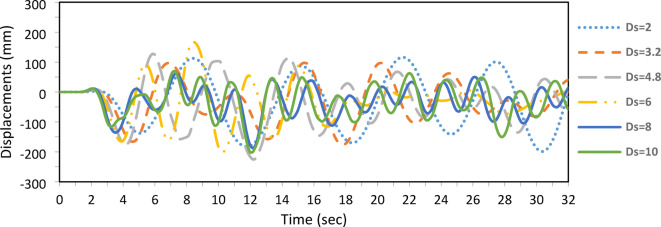
Dynamic responses of PSCs under EI-Centro Waves corresponding to cable diameter.

For the seismic analysis, increasing the stay diameter reduces peak displacements up to a certain limit, beyond which further increases in diameter and prestressing force become negligible. This confirms that beyond a specific threshold, increasing the stay diameter and corresponding prestressing force does not enhance the static or dynamic performance of the PSCs shown in Fig. 13.

### Geometry configuration

The overall geometric configuration, including the number of cross-arm levels, significantly influences the buckling loads and seismic response of PSCs. This study examines configurations with one, two, and three cross-arm levels, as shown in Fig. 2. The column length is set to 10,000 mm to accommodate the three-level configuration, with a main cross-arm length of 1,000 mm (2a/L = 0.2). For the two-level configuration, cross-arms are placed 3,000 mm from both ends, spaced 4,000 mm apart. In the three-level configuration, the column is evenly divided (L/4), and the secondary arms are 750 mm (75% of the main arm’s length). The cable diameter is 9.6 mm with four branches, and the cross-sectional and material parameters are ${D}_{co}$ = 76.2 mm, ${D}_{ci}$= 63.5 mm, ${D}_{ao}$ = 76.2mm, ${D}_{ai}$ = 63.5 mm, ${E}_{c}$ = 201 kN/mm^2^, ${E}_{a}$= 201 kN/mm^2^, and ${E}_{s}$ = 202 kN/mm^2^.

In the buckling analysis, two levels of cross-arms demonstrate higher buckling loads compared to one and three levels, as shown in Table 3. Optimal prestressing force (${T}_{opt}$) is applied, corresponding to the maximum buckling load. It is observed that, for the three-level configuration, the maximum prestressing force is achieved when both central and side cables become fully active, eliminating slack.

**Table 3:** Static buckling force with No cross-arm levels and T_opt_.

No of levels	${T}_{opt}$ (kN)	Buckling force (kN)
One level	8.78	129.45
Two levels	17.60	306.61
Three levels	14.03	282.95

**Fig. 14:**
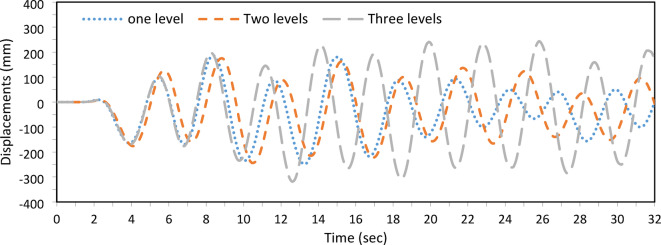
Dynamic responses of PSCs under EI-Centro Waves for one-, two-, and three-levels of cross-arms.

In the dynamic analysis, one- and two-level configurations, with all cables active after initial tension, show superior performance by reducing peak and maximum displacements (Fig. 14). The three-level configuration exhibits the highest displacement amplitude, suggesting greater susceptibility to dynamic forces. The oscillation frequency remains consistent across all configurations, indicating similar responses to the primary frequency content of the El-Centro seismic waves. However, slight phase differences are observed, with the one- and two-level configurations closely aligned, while the three-level configuration shows a noticeable phase shift.

### Number of cross-arm branches

**Fig. 15:**
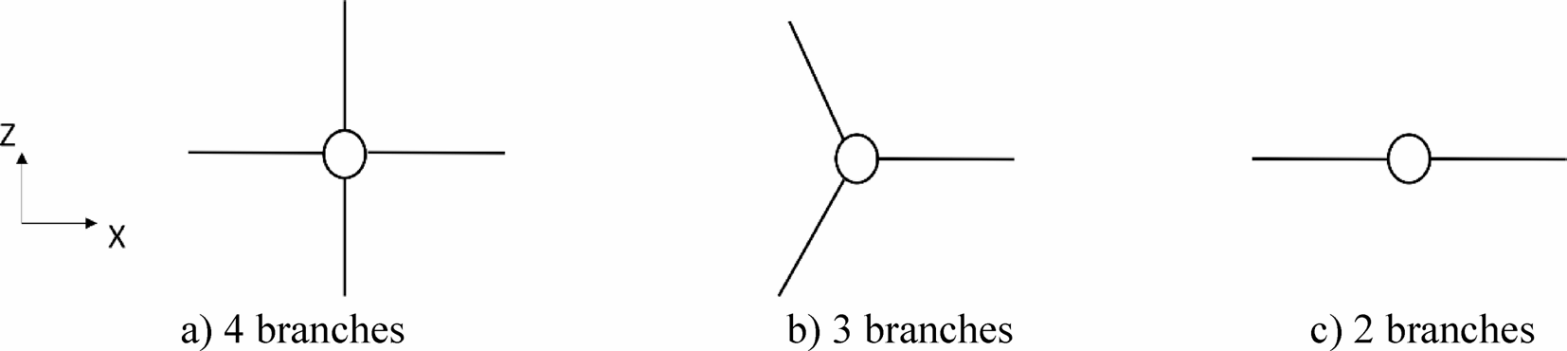
Different numbers of branches in the cross-arm system.

In this example, PSCs with three distinct cross-arm configurations, featuring 2, 3, or 4 branches, are investigated, as shown in Fig. 15. The prestressing cables are applied to selectively influence the targeted buckling mode by restraining out-of-plane buckling in configurations with two branches. The model parameters are as follows: main column length of 3050 mm, cross-arm length of 305 mm, and cable diameter of 4.8 mm, with a single cross-arm level (Fig. 15). The additional dimensions and material properties include: outer column diameter ${D}_{co}$= 38.1 mm, ${D}_{ci}$ = 25.4 mm, ${D}_{ao}$ = 38.1 mm, ${D}_{ai}$ = 25.4 mm, ${E}_{c}$ = 201 kN/mm^2^, ${E}_{a}$= 201 kN/mm^2^, and ${E}_{s}$ = 202 kN/mm^2^.

The analysis of static and dynamic responses under various loading conditions demonstrated that the number of cross-arm branches, illustrated in Fig. 15, significantly affects the buckling load, as detailed in Table 4. Specifically, the three-branch configuration increased the buckling load from 68.26 kN (observed with the two-branch configuration) to 108.12 kN, indicating a substantial gain in load capacity. In contrast, the four-branch configuration only slightly improved over the three-branch setup. Therefore, the three-branch configuration is recommended for standalone PSCs, offering an optimal balance between structural efficiency and load-carrying capacity.

**Table 4:** Static buckling force with No cross-arm branches and T_opt._.

No of branches	${T}_{opt}$ (kN)	Buckling force (kN)
2 branches	3.49	68.26
3 branches	4.36	108.12
4 branches	4.36	114.33

**Fig. 16:**
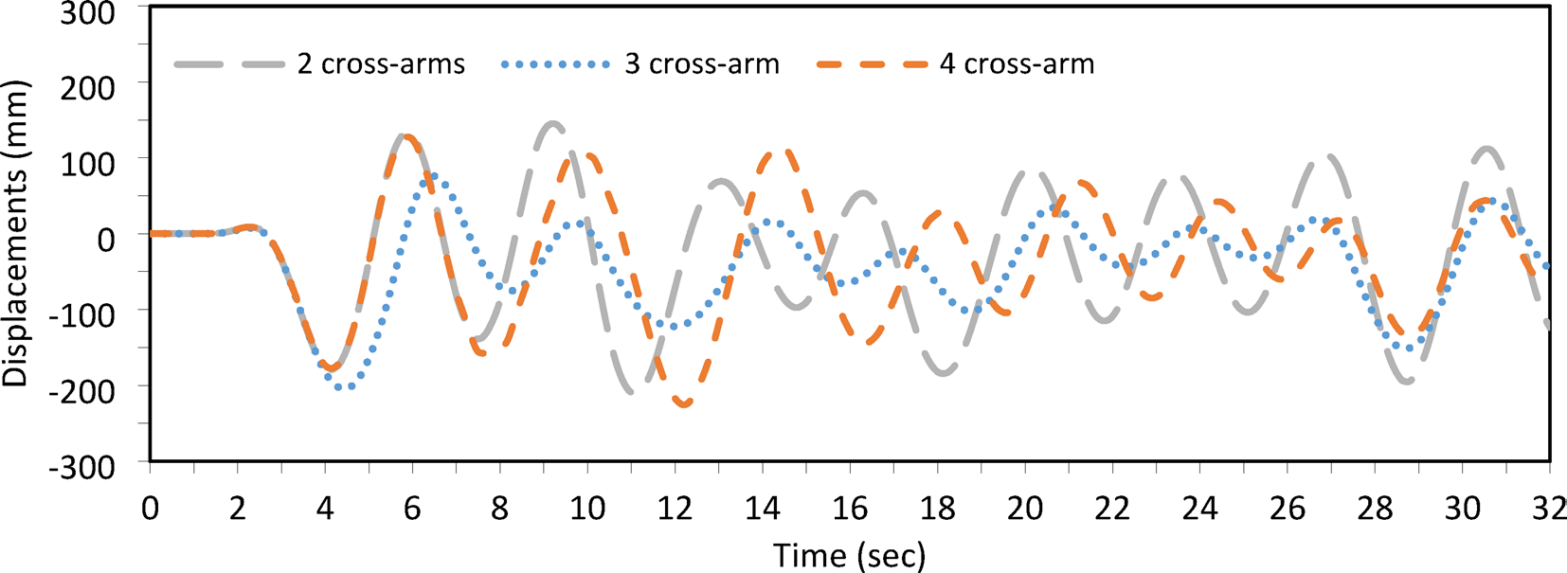
Dynamic responses of a PSC under EI-Centro Waves with No. of cross-arm branches.

On the other hand, the dynamic responses of the three configurations were evaluated under identical modeling parameters, applying El Centro seismic waves. Among the configurations, the three-branch cross-arm setup exhibited the lowest displacement amplitude, indicating a reduced susceptibility to dynamic forces. This finding suggests that increasing the number of cross-arm branches beyond three does not substantially enhance the static or dynamic performance of the PSCs, as illustrated in Fig. 16. Consequently, the three-branch configuration remains optimal for achieving stability and resilience under both static and dynamic conditions.

## Design guidelines

This section presents design guidelines and refined equations to predict PSCs load-bearing capacity with two-level cross-arms. Wadee et al.^36^ proposed a method to determine the ultimate capacity of PSCs, based on curve-fitting experimental and numerical simulation results. These equations predict the ultimate capacity ${N}_{EQ}$ of stayed columns for symmetric and antisymmetric buckling modes, requiring the calculation of elastic critical buckling loads. Wadee’s method was previously validated against experimental data from M. Serra’s research on long columns^13^. Available analytical methods for determining elastic buckling loads and load-carrying capacity were also compared with experimental results to verify their accuracy^13^.

For one-level prestressed stayed columns, the critical buckling load is determined by the interaction between the stays, cross-arms, and the column itself. The critical buckling load is calculated using the following equations.

10 $$N^{c} = C_{2 } \left( {N_{\max }^{c} - 2nT_{i } \cos \alpha } \right)$$

11 $$\left( {\frac{{N_{Eq} }}{{N^{c} }}} \right)sym_{Z3} = \left[ {\frac{{\left( {\frac{{N_{Eq} }}{{N^{c} }}} \right)_{{3T_{opt} }} - \left( {\frac{{N_{Eq} }}{{N^{c} }}} \right)_{{T_{opt} }} }}{{2T_{opt} }}} \right]\left( {T - T_{opt} } \right) + \left( {\frac{{N_{Eq} }}{{N^{c} }}} \right)_{{T_{opt} }}$$

12 $$\left( {\frac{{N_{Eq} }}{{N^{c} }}} \right)_{{3T_{opt} }} = 1 - 1.2\left( {\frac{2a}{{L_{c} }}} \right)\quad for\;\frac{L}{1000}$$

13 $$\left( {\frac{{N_{Eq} }}{{N^{c} }}} \right)_{{T_{opt} }} = 14\left( {\frac{2a}{{L_{c} }}} \right)^{2} - 3.1\left( {\frac{2a}{{L_{c} }}} \right) + 0.75\quad for\;\frac{L}{1000}$$

where ${C}_{2}$ is the stiffness factor, ${N}_{max}^{c}$ is the maximum critical buckling load, ${T}_{i}$ is the initial prestress, $\alpha$ is the angle between the stays and the column, and $n$ is the number of stays per cross-arm. These equations reliably estimate buckling loads for columns with one level of cross-arm.

The proposed FE models were applied to simulate PSCs with a single level of cross-arm, with a column length ${L}_{c}=12000$ mm. Geometric configurations and column dimensions used for comparison are detailed in Table 5. Simulation outcomes were compared with both experimental data and existing equations for ultimate load-carrying capacity, as summarized in Table 6^13^. This comparison revealed a mean ratio of 1.11, with a coefficient of variation (COV) of 0.19, while the FE simulation-to-test ratio was 0.95, with a COV of 0.13, as shown in Table 6. These results confirm the consistency between the proposed FE-based outcomes, and load predictions from Eqs. (11–13), and experimental test results.

**Table 5:** Summary of geometric measurements.

Column	${D}_{co}$ (mm)	${D}_{ci}$ (mm)	${D}_{ao}$ (mm)	${D}_{ai}$ (mm)	a (mm)
CO1	101.84	85.3	101.65	85.296	590.74
CO2	101.58	85.6	101.57	85.226	589.53
CO3	139.62	126.34	101.27	84.976	579.847
CO4	139.82	126.08	101.625	85.421	583.155

**Table 6:** Summary of test results and analytical calculations.

Col	Cable (mm)	Mode	${T}_{i}$ (kN)	${N}_{Test}$ (kN)^13^	${N}_{Eq}$ Eq. (11)^13^	${N}_{FE}$ (kN) Present study	${N}_{FE}/{N}_{Eq}$	${N}_{FE}/{N}_{Test}$
CO1	10	1	2	106.23	78.91	86.98	1.10	0.82
		1	4	134.36	101.18	107.82	1.06	0.80
		3	7.5	188.87	129.18	128.73	0.99	0.68
		3	9	163.71	137.5	127.26	0.92	0.78
		1	10.5	149.91	143	116.48	0.81	0.78
	13	1	2	112.17	93.07	115.39	1.24	1.03
		2	4	144.14	104.11	184.91	1.77	1.28
		3	7.5	178.04	123.83	187.30	1.51	1.05
		3	9	173.32	130.35	183.92	1.41	1.06
		3	10.5	193.24	135.72	179.88	1.32	0.93
CO2	10	3	2	123.52	75.68	86.66	1.14	0.70
		3	4	128.24	96.18	107.47	1.12	0.84
		3	7.5	124.92	121.78	126.44	1.04	1.012
		3	9	128.07	128.75	124.94	0.97	0.97
		3	10.5	129.12	144.4	121.71	0.84	0.94
	13	1	2	120.2	89.83	105.77	1.18	0.88
		1	4	185.37	100.22	113.51	1.13	0.61
		3	7.5	154.8	118.11	141.48	1.19	0.91
		3	9	156.9	123.92	147.12	1.18	0.98
		3	10.5	163.71	128.62	150.02	1.16	0.92
CO3	10	1	2	129.47	103.59	132.15	1.27	1.02
		1	4	148.51	130.75	147.17	1.13	0.99
		1	7.5	157.42	169.86	167.53	0.98	1.06
		1	10.5	185.72	194.87	168.95	0.86	0.91
		1	14	192.54	214.12	168.98	0.79	0.88
	13	1	2	150.78	189.54	157.09	0.83	1.04
		1	4	169.3	131.4	163.74	1.25	0.97
		1	7.5	175.42	156.49	190.72	1.22	1.09
		1	10.5	203.54	173.79	209.99	1.21	1.03
		1	14	233.42	189.07	219.72	1.16	0.94
CO4	10	1	2	132.26	107.79	135.60	1.26	1.02
		1	4	144.84	136.69	150.45	1.10	1.04
		1	7.5	167.38	178.74	171.33	0.96	1.02
		1	10.5	177.51	206.14	173.24	0.84	0.97
		1	14	189.74	228	173.04	0.76	0.91
	13	1	2	157.59	197.09	161.03	0.82	1.02
		1	4	164.58	136.18	167.67	1.23	1.02
		1	7.5	189.22	163.08	194.81	1.19	1.03
		1	10.5	199.7	181.84	214.4	1.18	1.07
		1	14	252.29	198.75	224.362	1.13	0.89
						Mean	1.11	0.95
						COV	0.19	0.13

This study extends the existing equations for one-level stayed columns to accurately predict the buckling load of two-level stayed columns, as shown in Fig. 2b. Using FE analyses and curve fitting techniques for Mode 1 (symmetric), two-level stayed columns are developed with a length of ${L}_{c}=12000$ mm and an initial tension of ${T}_{i }=4$ kN. These analyses allowed us to describe the behavior of the critical parameter ${C}_{2}$, which governs buckling behavior. The column has the following properties: ${D}_{s}=10$ mm, ${D}_{co}$= 101.84 mm, ${D}_{ci}$ = 85.3 mm, ${D}_{ao}$ = 101.65 mm, ${D}_{ai}$ = 85.266 mm, ${E}_{c}$ = 201 kN/mm^2^, ${E}_{a}$= 201 kN/mm^2^, and ${E}_{s}$ = 100 kN/mm^2^.

**Fig. 17:**
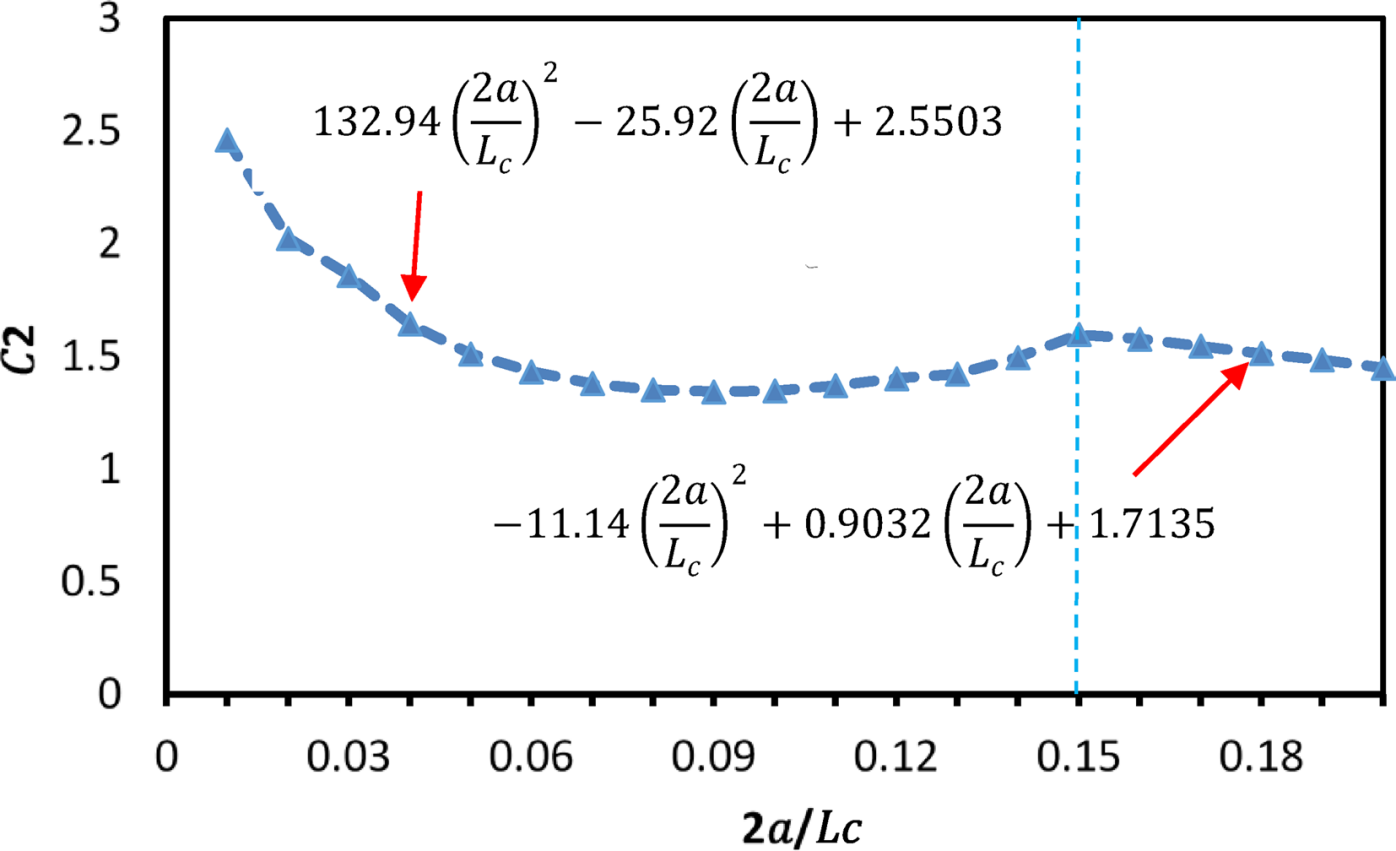
Relation between 2*a*/*Lc* and *C*2.

The curve fitting was performed for normalized cross-arm lengths $2a/{L}_{c}$ between 0.01 and 0.2, using FE models, each with a geometric imperfection of ${L}_{c}/1000$. The analysis revealed that the relationship between ${C}_{2}$ and the ratio $2a/{L}_{c}$ is nonlinear and can be divided into two distinct regions, as shown in Fig. 17:

- For $2a/{L}_{c}\le$ 0.15: ${C}_{2}$ exhibits a complex variation, initially decreasing with increasing $2a/{L}_{c}$, reaching a minimum, and then slightly increasing.
- For $2a/{L}_{c}>$ 0.15: ${C}_{2}$ steadily declines as the cross-arm spacing increases.

The proposed approach for two-level PSCs applies Eqs. (10)–(13) and introduces a newly defined parameter, ${C}_{2}$, derived from curve fitting of FE results, as illustrated in Fig. 17. The modified equations are presented below:

14 $$C_{{2\left( {new} \right) }} = 132.94\left( {\frac{2a}{{L_{c} }}} \right)^{2} - 25.92\left( {\frac{2a}{{L_{c} }}} \right) + 2.5503\quad \frac{2a}{{L_{c} }} \le 0.15$$

15 $$C_{{2\left( {new} \right) }} = - 11.14\left( {\frac{2a}{{L_{c} }}} \right)^{2} + 0.9032\left( {\frac{2a}{{L_{c} }}} \right) + 1.7135\quad \frac{2a}{{L_{c} }} > 0.15$$

To ensure the validity and reliability of these proposed equations, the load-carrying capacity was calculated using this approach and compared to FE model results. The column lengths were set to 10,000 mm and 12,000 mm, with corresponding initial tensions ${T}_{i}$ of 7.5 kN and 4 kN, respectively. The predicted-to-FE ratios for load-carrying capacity averaged 0.99 and 1.00, with coefficients of variation (COV) of 0.03 and 0.04, respectively, as shown in Tables 7 and 8. These results confirm that the proposed equations accurately predict the load-carrying capacity for the two-level PSC system.

**Table 7:** Load carrying capacities of PSC with a length of 10,000 mm (predicted and FE results).

No.	$\frac{2a}{{L}_{c}}$	A (mm)	${N}_{FE}$ (kN)	${T}_{opt}$ (kN)	${Nc(}_{max})$ (kN)	${C2}_{(new)}$	${Nc(}_{Eq})$ (kN)	${N}_{Eq}$ (kN)	${N}_{Eq}/{N}_{FE}$
1	0.01	50	54.25	0.76	56.23	2.30	60.45	50.8	0.94
2	0.02	100	58.17	0.88	65.09	2.09	73.20	59.89	1.03
3	0.03	150	68.55	1.02	76.26	1.89	87.59	69.78	1.02
4	0.04	200	82.37	1.27	94.65	1.73	111.70	86.52	1.05
5	0.05	250	99.11	1.56	117.08	1.59	138.31	104.1	1.05
6	0.06	300	118.46	1.91	143.03	1.47	166.75	122	1.03
7	0.07	350	140.2	2.3	172.81	1.39	198.36	141	1.01
8	0.08	400	163.99	2.72	205.42	1.33	233.13	161.1	0.98
9	0.09	450	189.56	3.16	239.37	1.29	271.34	182.7	0.96
10	0.1	500	216.53	3.6	274.02	1.29	314.66	207	0.96
11	0.11	550	244.5	4.01	306.02	1.31	361.46	233.4	0.95
12	0.12	600	273.37	4.37	335.59	1.35	414.48	264.1	0.97
13	0.13	650	301.81	4.74	365.80	1.43	480.15	303.2	1.00
14	0.14	700	330.27	4.88	378.62	1.53	533.36	337.4	1.02
15	0.15	750	369.97	5.04	393.54	1.60	582.22	370.3	1.00
16	0.16	800	379.02	5.13	403.01	1.57	587.98	378.2	0.99
17	0.17	850	387.06	5.22	412.59	1.55	592.58	387.1	1.00
18	0.18	900	394.15	5.28	420.34	1.52	592.99	395	1.00
19	0.19	950	400.4	5.29	424.24	1.48	586.34	399.9	0.99
20	0.2	1000	402.26	5.26	425.22	1.45	574.32	402.3	1.00
								Mean	0.99
								COV	0.03

**Table 8:** Load carrying capacities of PSC with a length of 12,000 mm (predicted and FE results).

No.	$\frac{2a}{{L}_{c}}$	A (mm)	${N}_{FE}$ (kN)	${T}_{opt}$ (kN)	${Nc(}_{max})$ (kN)	${C2}_{(new)}$	${Nc(}_{Eq})$ (kN)	${N}_{Eq}$ (kN)	${N}_{Eq}/{N}_{FE}$
1	0.01	60	37.625	0.47	35.19	2.30	44.23	37.08	0.99
2	0.02	120	41.889	0.55	41.30	2.06	52.77	43.02	1.02
3	0.03	180	50.385	0.68	50.67	1.89	65.65	51.98	1.03
4	0.04	240	61.975	0.88	65.73	1.77	85.89	65.87	1.06
5	0.05	300	75.586	1.11	82.74	1.59	105.96	78.64	1.04
6	0.06	360	91.029	1.41	105.73	1.47	132.33	94.74	1.04
7	0.07	420	107.89	1.81	136.12	1.39	166.76	114.7	1.06
8	0.08	480	125.45	2.12	160.16	1.33	191.52	127.3	1.01
9	0.09	540	143.14	2.49	188.33	1.29	223.24	143.2	1.00
10	0.1	600	160.58	2.86	217.62	1.29	259.85	161.8	1.01
11	0.11	660	177.16	3.28	245.26	1.31	300.08	181.4	1.02
12	0.12	720	192.46	3.43	262.93	1.35	334.74	200.7	1.04
13	0.13	780	238.03	3.61	278.55	1.43	375.20	223.7	0.94
14	0.14	840	265.51	3.68	285.85	1.53	412.62	247.5	0.93
15	0.15	900	272.06	3.71	289.21	1.60	437.30	265.9	0.98
16	0.16	960	277.68	3.73	293.26	1.57	436.77	270.3	0.97
17	0.17	1020	282.54	3.74	295.97	1.55	433.35	274.2	0.97
18	0.18	1080	288.08	3.75	298.70	1.52	429.17	278.9	0.97
19	0.19	1140	290.41	3.77	302.82	1.48	426.24	285.3	0.98
20	0.2	1200	293.47	3.81	308.35	1.45	424.45	293.6	1.00
								Mean	1.00
								COV	0.04

## Conclusions and summary

This paper investigates the static and dynamic behavior of PSCs, with a particular focus on buckling and dynamic responses, through FE analysis and parametric studies. A validated FE protocol was developed using scripting techniques, with analytical and experimental results serving as benchmarks for verification. A comprehensive parametric study was conducted, examining critical factors such as cross-arm length, cable diameter, and geometric configurations. Finally, design guidelines and equations are provided for predicting the load-bearing capacity of PSCs with two levels of cross-arms. The key findings are as follows:

- The study demonstrates that increasing cross-arm length and cable diameter enhances buckling resistance up to an optimal parameter (2a/L = 0.2). Beyond this limit, further increases yield diminishing benefits.
- In multi-level cross-arm configurations, it was found that achieving higher initial tension in all cables does not substantially enhance buckling loads or reduce dynamic displacements.
- The three-branch cross-arm configuration showed the best balance of static and dynamic performance, enhancing buckling resistance and reducing peak displacements. While similar systems have been studied as beam-columns under combined loading, this work explores their potential in roof structures, highlighting dynamic advantages and identifying areas needing further validation.
- A newly proposed parameter ${C}_{2}$, derived from curve fitting based on FE simulations of PSCs, has been integrated into the existing equations for predicting the load-carrying capacity of columns with two levels of cross-arms. Comparisons with FE results demonstrate the accuracy and effectiveness of this enhanced approach in improving predictive reliability.
- While the structural performance of PSCs is important, sustainability is an additional consideration in modern structural engineering practice. PSCs offer material efficiency by using prestressing to improve buckling resistance while maintaining a lightweight cross-arm configuration and a smaller cross section. Consequently, PSCs could be considered more effective at reducing embodied carbon emissions associated with steel production compared to conventional systems.

This study improves PSCs behavior understanding by integrating new design parameters into existing methodologies, bridging theory and practice. It offers refined design equations and practical recommendations for optimizing PSCs performance. Additionally, because PSCs systems can potentially be modular and reused, they have the potential to offer a more sustainable option in construction. Future work could involve diverse seismic records, varying intensity, direction, and frequency content, to further assess the system’s dynamic behavior. Also, a life cycle assessment (LCA) that quantifies the environmental implications of PSCs systems (carbon footprint, energy consumption in steel production, end-of-life recyclability, etc.) to educate and influence environmentally informed design practices.

## Data Availability

The datasets used and/or analyzed during the current study available from the corresponding author on reasonable request.
